# Contribution and Regulation of HIF-1α in Testicular Injury Induced by Diabetes Mellitus

**DOI:** 10.3390/biom15081190

**Published:** 2025-08-19

**Authors:** Defan Wang, Zhenghong Zhang, Renfeng Xu, Zhengchao Wang

**Affiliations:** 1 Fujian Provincial Key Laboratory of Reproductive Health Research, School of Medicine, Xiamen University, Xiamen 361102, China; defanwang2021@gmail.com (D.W.); xurenfeng131772@gmail.com (R.X.); 2 Provincial Key Laboratory for Developmental Biology and Neurosciences, College of Life Sciences, Fujian Normal University, Fuzhou 350007, China; zhangzh@fjnu.edu.cn; 3 Department of Pharmacological and Pharmaceutical Sciences, College of Pharmacy, University of Houston, Houston, TX 77204, USA

**Keywords:** diabetes mellitus, testicular injury, hypoxia-inducible factor-1α, oxidative stress, Leydig cells, targeted therapy

## Abstract

Diabetes mellitus, as a metabolic disorder, has received growing attention for its detrimental effects on the male reproductive system (particularly the testes) manifesting as increased oxidative stress, reduced blood perfusion, heightened inflammation, and germ cell apoptosis under hyperglycemic conditions. Hypoxia-inducible factor (HIF)-1α, a pivotal transcription factor in cellular hypoxia responses, plays a crucial role in regulating metabolism, angiogenesis, and apoptosis. Emerging evidence underscores its significant physiological and pathological roles in diabetic testicular injury. This review outlines the structural domains, activation mechanisms, and key target genes of HIF-1α, and further examines its involvement in diabetes-induced oxidative stress, impaired perfusion, endocrine dysregulation, and the imbalance of apoptosis and autophagy in testicular tissue. Notably, HIF-1α exerts protective effects by activating canonical signaling pathways such as phosphoinositide-3 kinase (PI-3K)/protein kinase B (Akt), mitogen-activated protein kinase (MAPK)/extracellular signal-regulated kinase (ERK), and nuclear factor (NF)-κB, thereby enhancing antioxidant gene expression, promoting angiogenesis, and upregulating anti-apoptotic proteins. Furthermore, HIF-1α may help stabilize androgen levels by preserving Leydig cell function, potentially alleviating diabetes-associated gonadal dysfunction. This review also discusses the feasibility of targeting HIF-1α as a novel therapeutic strategy. In conclusion, a comprehensive understanding of HIF-1α’s mechanistic role in diabetic testicular damage provides valuable insights into the pathogenesis of diabetes-related reproductive disorders and offers new avenues for therapeutic intervention.

## 1. Introduction

Diabetes has emerged as a major global public health concern, affecting hundreds of millions of individuals. With the aging population and shifts in lifestyle, the prevalence of diabetes is expected to rise steadily in the coming years. Persistent hyperglycemia not only compromises the cardiovascular, renal, and nervous systems [1,2], but also exerts significant adverse effects on the male reproductive system [3,4]. Studies have shown that men with diabetes frequently exhibit pathological features including reduced testicular volume [5], impaired spermatogenesis [6], hormonal imbalances [7], and decreased fertility [8]. These abnormalities are closely associated with increased germ cell apoptosis, diminished local blood flow, heightened oxidative stress, and chronic inflammation [5,6,7,8]. Among the molecular mediators linking diabetes and reproductive dysfunction, HIF-1α plays a particularly pivotal role [9,10,11]. 

The testis is characterized by a relatively hypoxic microenvironment. HIF-1α promotes germ cell survival under conditions of reduced oxygen availability. Takahashi et al. reported that HIF-1α is expressed during early embryonic development, including the two-cell stage, morula, and blastocyst, and remains highly expressed in spermatogonia of adult male testes [12]. Powell et al. demonstrated that HIF-1α protein levels increased markedly after 15 min of ischemia and remained elevated from 30 min to 6 h in a testicular ischemia model, suggesting that its rapid activation plays a protective role against ischemic damage in testicular tissue [13].

HIF-1α is a central transcription factor involved in cellular adaptation to hypoxic conditions [14]. Beyond its role in regulating cellular metabolism and energy homeostasis [15], HIF-1α modulates apoptosis [16,17], angiogenesis [18], and inflammatory responses [19], underscoring its relevance in metabolic disorders, cardiovascular diseases, and tissue repair processes [20,21,22]. For example, Basheeruddin and Qausain demonstrated that in testicular cells, HIF-1α enhances cellular tolerance to hypoxia by upregulating glycolysis-related genes such as glucose transporter-1 (GLUT-1) and lactate dehydrogenase A (LDHA), thereby sustaining energy metabolism and mitigating hypoxia-induced cellular injury [23]. Similarly, Yang et al. found that HIF-1α not only promotes angiogenesis and cell survival via vascular endothelial growth factor (VEGF) but also enhances the cellular self-repair capacity under stress by regulating autophagy-related genes [24]. Notably, metabolic disturbances in diabetes can induce tissue hypoxia, and subsequent activation of HIF-1α may contribute to testicular dysfunction [9,10]. HIF-1α may influence germ cell viability by modulating survival and apoptotic pathways [25]. However, its specific role in diabetic testicular injury remains incompletely understood, and its bidirectional regulatory potential in testicular injury and repair warrants systematic investigation.

This review aims to explore the regulatory role of HIF-1α in diabetes-induced testicular damage. By synthesizing recent research advances, it further analyzes the role of HIF-1α in testicular oxidative stress, vascular function, apoptosis, and endocrine regulation, and elucidates the involvement of associated signaling pathways. Additionally, the review discusses the feasibility and clinical potential of therapeutic strategies targeting HIF-1α for the treatment of diabetic testicular injury.

The literature discussed in this review was identified through a systematic search of PubMed, Web of Science, and Scopus databases up to June 2025 using keywords such as ‘HIF-1α’, ‘diabetes’, ‘testicular injury’, ‘male fertility’, and ‘hypoxia signaling’. Inclusion criteria were peer-reviewed original research articles or reviews in English that explored the role of HIF-1α in diabetic or testicular contexts. Studies unrelated to male reproductive physiology, not involving HIF-1α modulation, or focused exclusively on non-mammalian species were excluded.

## 2. The Structure and Biological Function of HIF-1α

**Figure 1:**
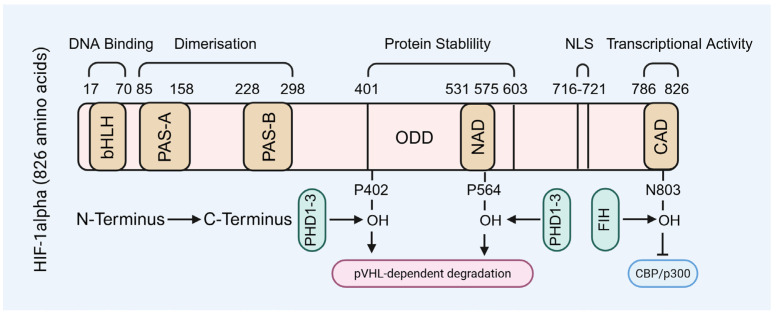
The molecular structure of HIF-1α subunit. NLS: nuclear localization signal.

HIF-1 is a HIF-1α/HIF-1β heterodimer, with HIF-1α serving as the oxygen-sensitive regulatory subunit. HIF-1α contains an n-terminal basic helix-loop-helix (bHLH) domain, a c-terminal activation domain, and an oxygen-sensing domain [10,26,27,28,29,30,31]. The bHLH domain is the structural core of HIF-1α, comprising two α-helices connected by a loop, enabling HIF-1α to bind hypoxia-responsive elements in target gene promoters (Figure 1). Adjacent to this domain, HIF-1α also possesses a Per-ARNT-Sim (PAS) domain, which is commonly found in transcription factors and facilitates protein–protein interactions essential for transcriptional complex assembly (Figure 1). HIF-1α contains multiple hydroxylation sites, primarily at proline residues; under normoxic conditions, hydroxylation at these sites targets HIF-1α for proteasomal degradation, whereas hypoxia inhibits hydroxylation, thereby stabilizing HIF-1α and enhancing its transcriptional activity (Figure 1). Additionally, the C-terminal activation domain recruits transcriptional coactivators such as CREB-binding protein (CBP)/p300, which are critical for initiating the transcription of hypoxia-responsive genes (Figure 1). Collectively, these domains are essential for the transcriptional regulatory function of HIF-1α [10,26,27,28,29,30,31].

**Figure 2:**
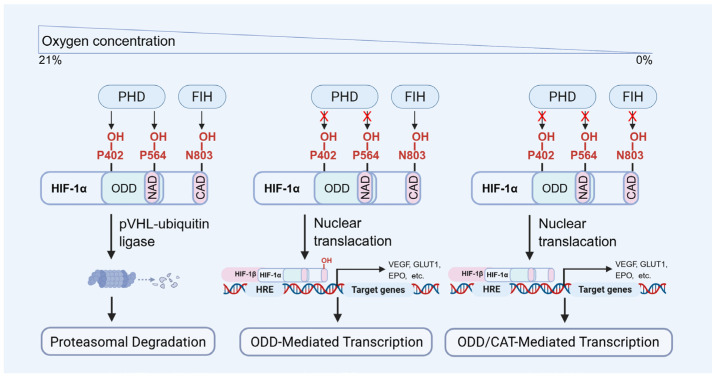
The molecular regulation of HIF-1α degradation and transcriptional activity. PHD: prolyl hydroxylase domain proteins, FIH: factor inhibiting HIF.

Under normoxic conditions, HIF-1α is hydroxylated by prolyl hydroxylases (PHDs) and subsequently recognized by the von Hippel–Lindau protein (VHL, Figure 2), which mediates its ubiquitin-dependent proteasomal degradation [23,32,33,34,35]. While under hypoxic conditions, HIF-1α escapes hydroxylation, becomes stabilized, translocated into the nucleus, and dimerizes with HIF-1β to form an active transcriptional complex (Figure 2). This complex binds to hypoxia response elements (HREs) of target genes, thereby initiating their transcription and promoting adaptive cellular responses, including angiogenesis, enhanced glycolysis, and survival signaling. The principal function of HIF-1α is to facilitate cellular adaptation to hypoxia by regulating metabolism and promoting cell viability across diverse tissue types [23,36,37,38]. Its transcriptional targets include VEGF, erythropoietin (EPO), GLUT-1, and acetyl-CoA synthetase, among others. The regulatory scope of HIF-1α encompasses diverse biological processes, including metabolic reprogramming, neovascularization, anti-apoptotic signaling, autophagy activation, and modulation of inflammatory responses [23,31,39].

With the rising global prevalence of diabetes, the role of HIF-1α in diabetes-induced testicular injury has garnered increasing research interest. Spermatogenic cells require substantial energy during development, which is predominantly generated through oxidative phosphorylation. Therefore, oxygen availability directly influences both the quality and quantity of sperm produced [40,41,42]. Hormone synthesis by testicular Leydig cells is also oxygen-dependent, as the biosynthesis of testosterone involves multiple enzymatic steps, each requiring adequate oxygen supply to sustain enzymatic efficiency and reaction fidelity [43]. In individuals with diabetes, tissue hypoxia is common and is primarily attributed to vascular dysfunction and elevated oxidative stress [44]. Diabetes induces microvascular complications, including endothelial injury in capillaries and altered hemodynamics, which collectively impair testicular perfusion and oxygen delivery [45]. Moreover, the diabetic state is characterized by heightened oxidative stress, thereby impairing cellular oxygen utilization efficiency. Studies have demonstrated that hyperglycemia-driven OS promotes ROS generation, leading to cellular damage and further compromising testicular function [10,28,46].

Thus, HIF-1α promotes metabolic pathways such as glycolysis and lactic acid fermentation, enabling cells to survive under hypoxic conditions, which is particularly critical in low-oxygen tissues like the testis [23]. However, in certain contexts, HIF-1α upregulation has been linked to diabetes-associated complications, suggesting that the regulation of HIF-1α may have a dual role in diabetes-related reproductive health issues [9].

## 3. The Mechanism of Diabetic Testicular Injury

**Figure 3:**
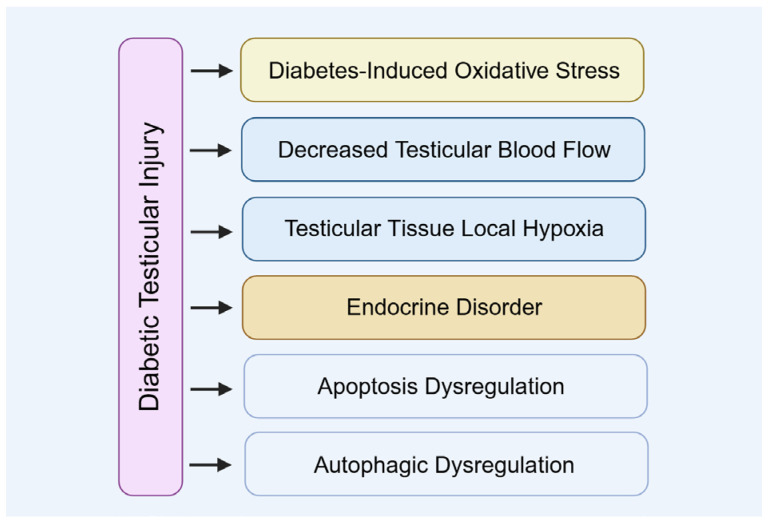
The mechanism of diabetic testicular injury.

The mechanism underlying diabetic testicular injury is a complex process involving multiple physiological alterations (Figure 3). Metabolic disturbances and microcirculatory impairments caused by diabetes directly compromise testicular health, subsequently disrupting testosterone synthesis and spermatogenesis.

### 3.1. Diabetes-Induced Oxidative Stress

**Figure 4:**
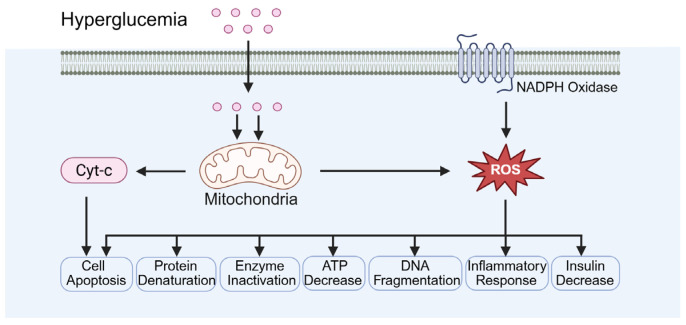
Diabetes-induced oxidative stress. Cyt-c: cytochrome. ROS: reactive oxygen species.

Oxidative stress results from an imbalance between free-radical generation and an antioxidant defense system (Figure 4). Hyperglycemia associated with diabetes can elevate oxidative stress levels, which is a key contributor to testicular damage. Minas et al. demonstrated that ROS induced by hyperglycemia directly damage the DNA of germ cells, causing DNA strand breaks and abnormal chromatin structure [47]. Biasi et al. reported that ROS impair mitochondrial function, decreasing ATP production, which adversely affects sperm motility and maturation [48]. Additionally, Kilarkaje et al. showed that ROS can induce apoptosis in supporting cells, further compromising spermatogenesis [49].

In addition, diabetes is associated with decreased expression of tissue antioxidant enzymes and increased NADPH oxidase activity (Figure 4). Iskender et al. reported that diabetic models exhibit a significant reduction in antioxidant enzymes, including SOD, CAT, and GPx, indicating the impairment of the antioxidant defense system [50]. Qiu et al. demonstrated that NADPH oxidase activity is markedly elevated in diabetic models, resulting in increased ROS production and exacerbation of oxidative stress [51]. Yoshikawa et al. observed significantly elevated ROS levels in the testicular tissue of diabetic rats accompanied by reduced antioxidant enzyme activity, which correlated with increased rates of cell apoptosis [52]. Furthermore, testicular tissue from diabetic rats exhibited disrupted collagen fiber organization and extracellular matrix accumulation, further compromising normal testicular function and spermatogenesis [52].

### 3.2. Decreased Testicular Blood Flow and Local Hypoxia

**Figure 5:**
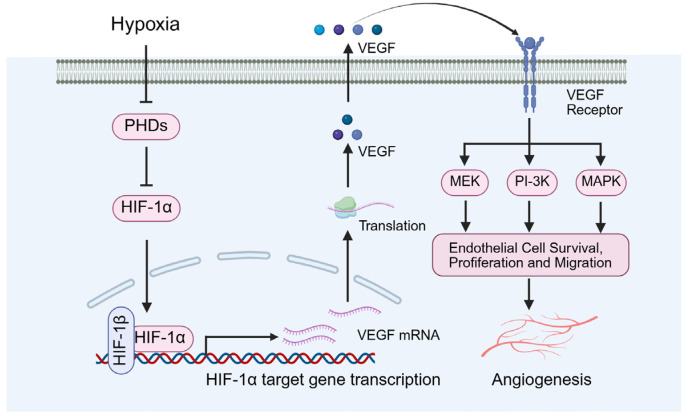
Diabetes-induced local hypoxia and angiogenesis. VEGF: vascular endothelial growth factor.

Testicular microcirculatory disturbance induced by diabetes is a critical mechanism contributing to impaired testicular function, with the reduction in VEGF identified as a primary factor (Figure 5). Goligorsky et al. reported that diabetic patients exhibit decreased microvascular density in testicular tissue accompanied by altered hemodynamics, resulting in localized hypoxia and inadequate nutrient supply [53]. Fagundes et al. demonstrated that this hypoxic environment inhibits the function of testicular interstitial cells, leading to reduced synthesis and secretion of testosterone [54]. Additionally, Alyami et al. observed significant downregulation of enzymes and genes involved in testosterone biosynthesis within the testicular tissue of diabetic patients [55].

Notably, Bowker et al. identified VEGF as a critical regulator of angiogenesis, promoting the proliferation and differentiation of endothelial cells, thereby preserving the normal structure and function of microvessels (Figure 5) [56]. Long et al. further demonstrated that VEGF is essential for maintaining adequate blood flow and nutrient delivery to the testes, with its reduction resulting in impaired testicular microcirculation and subsequent pathological alterations [57]. Specifically, decreased VEGF levels can precipitate testicular microcirculatory dysfunction, leading to ischemia and hypoxia within the testicular tissue. These changes contribute to testicular atrophy, disruption of the seminiferous tubule architecture, and impaired spermatogenesis [57].

In addition, under diabetic conditions, the upregulation of HIF-1α is generally regarded as an adaptive response that promotes angiogenesis by inducing the expression of genes such as VEGF (Figure 5), thereby enhancing tissue oxygenation [58,59]. However, chronic hyperglycemia may disrupt the normal function of HIF-1α, resulting in dysregulation of its signaling pathway [58,59].

### 3.3. Diabetes-Caused Endocrine Disorders

**Figure 6:**
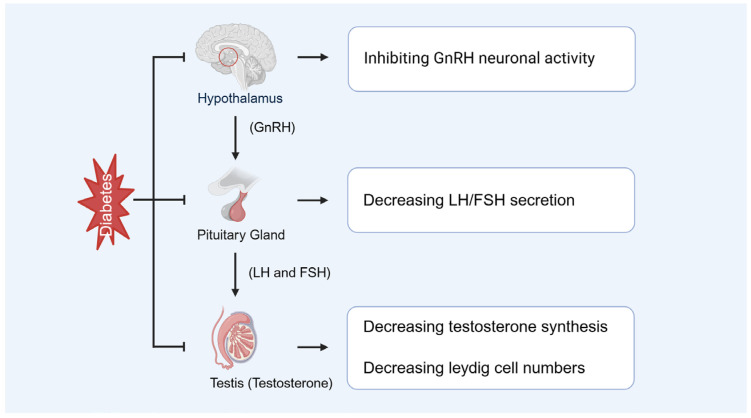
Diabetes-caused endocrine disorders. GnRH: gonadotropin-releasing hormone, LH: luteinizing hormone, FSH: follicle-stimulating hormone.

Diabetes-induced hyperglycemia suppresses testosterone production and causes hypogonadism, which is a key contributor to male infertility (Figure 6). Additionally, inflammatory responses in the male reproductive tract, often exacerbated under diabetic conditions, can impair spermatogenesis and reduce sperm count. These changes, together with coexisting andrological conditions such as varicocele or infection, may synergistically worsen testicular function in diabetic individuals. Musa and Graziani et al. demonstrated that reduced testosterone is closely associated with sexual dysfunction, diminished sperm quality, and decreased fertility [7,60]. Furthermore, Lotti and Facondo et al. indicated that low testosterone correlates significantly with reduced sperm concentration and total sperm count, thereby exacerbating male infertility [8,61]. Pelusi revealed that diabetes disrupts the hypothalamic-pituitary-gonadal (HPG) axis, causing hormonal imbalances that culminate in impaired testicular function, characterized by decreased testosterone production and defective spermatogenesis [62]. Additionally, Abd El-Twab et al. found significant downregulation of luteinizing hormone (LH) and follicle-stimulating hormone (FSH) receptor expression in the testes of diabetic patients, contributing to reduced testosterone synthesis [63].

Diabetes not only impairs Leydig cell function but may also lead to a reduction in their number (Figure 6). Wagner et al. demonstrated that in the testes of diabetic rats, the RNA expression of key steroidogenic enzymes, including Cyp11A1 and 3β-Hsd2, was significantly downregulated, resulting in decreased testosterone synthesis [64]. Mo et al. reported that diabetic mouse models exhibited a significant reduction in the number of fetal Leydig cells [65]. Furthermore, diabetes exacerbates Leydig cell loss by promoting apoptosis and inflammatory responses [64].

HIF-1α directly influences testosterone production in Leydig cells by regulating the expression of steroidogenic enzymes (Figure 6). Dhole et al. demonstrated that HIF-1α enhances steroid hormone and VEGF production through activation of downstream signaling pathways, including the cyclic adenosine monophosphate (cAMP)-protein kinase A (PKA) pathway [66]. Additionally, Sui et al. found that under hypoxic conditions, HIF-1α modulates steroidogenesis by regulating StAR expressions [67].

### 3.4. The Dysregulation of Apoptosis and Autophagy

**Figure 7:**
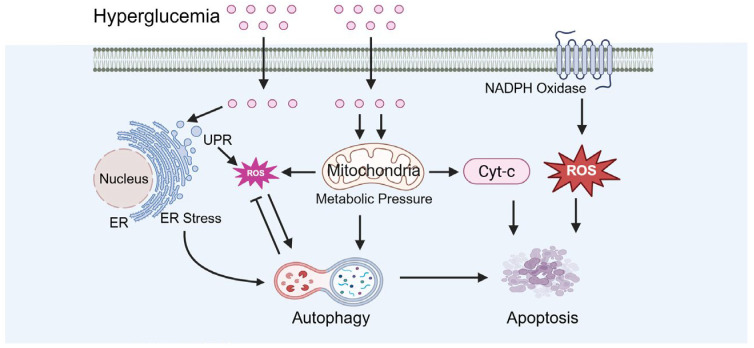
Diabetes-caused dysregulation of apoptosis and autophagy. ER: endoplasmic reticulum, UPR: unfolded protein response.

Under diabetic conditions, testicular cell apoptosis is increased while autophagy becomes dysregulated (Figure 7). Buhur and Du et al. observed an elevated Bax/Bcl-2 ratio and caspase-3 activation in diabetic models, indicating the induction of apoptotic pathways [68,69,70]. Tian et al. reported that autophagy exerts a dual role in diabetic testicular injury. Diabetes inhibits the PI-3K/AKT/mTOR signaling pathway, resulting in excessive autophagy activation that promotes apoptosis [71]. Conversely, clusterin overexpression mitigates diabetes-induced testicular damage by suppressing autophagy, as evidenced by decreased LC-3 and Beclin-1 levels, alongside reduced pro-apoptotic protein expressions like bax and caspase-3 [71].

Diabetes can induce chronic inflammatory responses that further damage testicular tissue, adversely affecting the survival and function of spermatogenic cells. Tvrdá et al. reported that in the testicular tissue of diabetic rats, excessive ROS production coupled with a reduction in antioxidant molecules caused oxidative damage to proteins and lipids, alongside a significant increase in proinflammatory cytokines, thereby exacerbating testicular dysfunction [72]. Similarly, Naderi et al. found that in a diabetic mouse model, the elevated levels of TNF-α/IL-6 were closely associated with the increased apoptosis of spermatogenic cells and the suppression of anti-inflammatory cytokines in the testis [73].

In addition, HIF-1α participates in apoptosis and autophagy in a time- and context-dependent manner, potentially exerting either protective or detrimental influences at different stages (Figure 7). Wang et al. demonstrated that, under hypoxic conditions, HIF-1α upregulates Bcl-2 while downregulating Bax, thereby inhibiting apoptosis [74]. Conversely, under certain pathological states, HIF-1α may promote apoptosis through activation of Bax and caspase-9 [74]. Furthermore, Pan and Abo et al. revealed that in hypoxic environments, HIF-1α can induce autophagy by upregulating Beclin-1, which contributes to cellular protection against damage [75,76].

## 4. The Role of HIF-1α in Diabetes-Induced Testicular Injury

**Figure 8:**
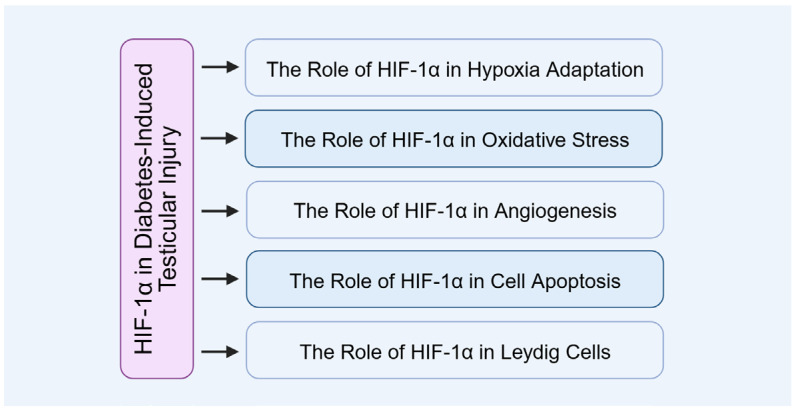
The role of HIF-1α in diabetes-induced testicular injury.

In diabetic testicular injury, the role of HIF-1α is especially significant. Its expression is markedly elevated in the testes of diabetic mice, functioning not only as a protective response to hypoxia and cellular stress but also closely associating with testicular microcirculatory dysfunction and impaired reproductive capacity (Figure 8). 

### 4.1. The Role of HIF-1α in Testicular Hypoxia Adaptation

**Figure 9:**
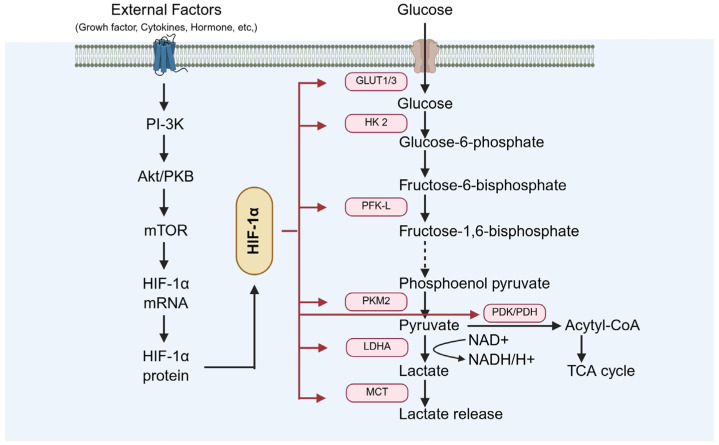
The role of HIF-1α in testicular hypoxia adaptation. GLUT-1: glucose transporter 1; LDHA: lactate dehydrogenase A.

Under diabetic conditions, activation of HIF-1 enhances testicular cell adaptability to hypoxia by improving glucose uptake and promoting metabolic reprogramming (Figure 9). Xiong et al. demonstrated that HIF-1α upregulates GLUT-1, thereby increasing cellular glucose uptake [77], and also regulates the expression of the glycolytic enzyme LDHA, facilitating metabolic adaptation under hypoxic conditions [77]. Furthermore, Liu ad Han et al. revealed that HIF-1α optimizes cellular energy metabolism and survival through PI-3K/AKT/mTOR signaling activation [15,78].

Besides metabolic reprogramming, HIF-1α-derived adaptive mechanisms also include the promotion of angiogenesis, anti-apoptotic and cytoprotective effects, and the regulation of Sertoli and Leydig cell functions. For example, Baset and Wang et al. demonstrated that HIF-1α upregulates VEGF, enhancing capillary density and oxygen delivery in the interstitial space, supporting the remodeling of existing microvasculature around seminiferous tubules [79,80]. Lui et al. demonstrated that in Sertoli cells, HIF-1α may maintain blood–testis barrier integrity via claudin-11 regulation under moderate hypoxia [81,82].

### 4.2. The Role of HIF-1α in Oxidative Stress Response

**Figure 10:**
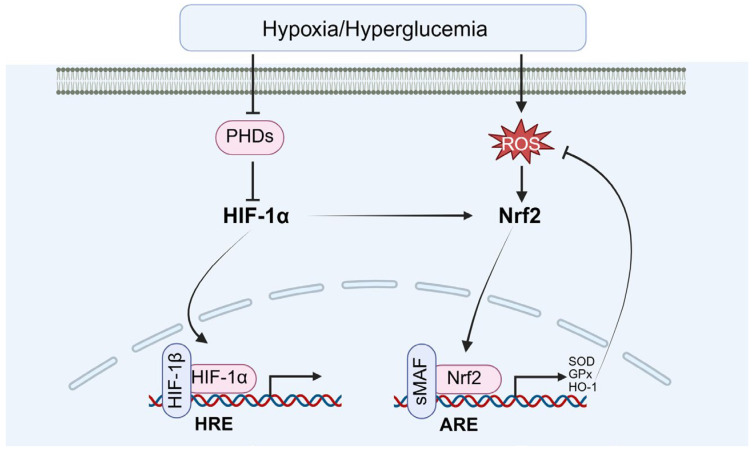
The role of HIF-1α in oxidative stress response. Nrf2: nuclear factor erythroid 2-related factor 2.

Under the heightened oxidative stress induced by diabetes, HIF-1α contributes to reducing oxidative damage in testicular cells by regulating antioxidant enzyme expressions, including SOD and GPx (Figure 10). Fang and Liu et al. demonstrated that HIF-1α activates antioxidant gene transcriptions via Nrf2 signaling pathway, thereby enhancing the testicular tissue’s capacity to scavenge ROS and providing cellular protection in a hyperglycemic environment [11,83]. Additionally, stabilization of HIF-1α further augments antioxidant defenses by upregulating uncoupling protein 2 (UCP2) expression [83].

Besides the induction of antioxidant defense, the mitochondrial quality control and redox-sensitive signaling cross-talk are also involved. For example, Hou and Wu et al. demonstrated that HIF-1α promotes mitophagy, selectively removing damaged, ROS-generating mitochondria by inducing B-cell leukemia/lymphoma 2/adenovirus E1B interacting protein 3 (BNIP3) and Nip-like protein X (NIX). This process limits oxidative injury and maintains cellular homeostasis [84,85]. Michalak K.P. and Michalak A.Z. demonstrated that HIF-1α can enhance Nrf2 activation, indirectly boosting transcription of a broad antioxidant gene repertoire. In parallel, it regulates Bcl-2 family proteins to preserve mitochondrial membrane integrity [86].

### 4.3. The Role of HIF-1α in Testicular Angiogenesis

**Figure 11:**
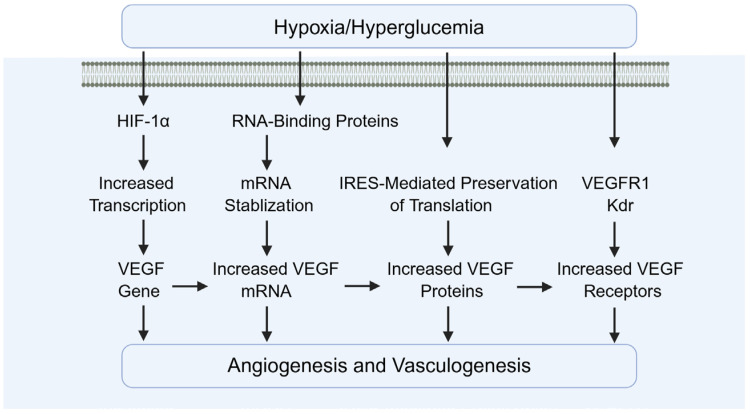
The role of HIF-1α in angiogenesis. VEGF: vascular endothelial growth factor.

Diabetes causes reduced testicular blood flow and disruption of microvascular architecture (Figure 11). As an upstream regulator of angiogenic factors such as VEGF, HIF-1α promotes testicular neovascularization and ameliorates local hypoxia. Wang et al. demonstrated that activation of HIF-1α upregulates angiogenic genes including VEGF and ANGPT1/2, leading to increased microvascular density and enhanced oxygen delivery in the testes. This improvement in microcirculation supports cellular survival and functional recovery [21,87,88,89]. 

In addition, Oda and Hsu et al. demonstrated that HIF-1α upregulates PlGF, promoting vessel sprouting and recruitment of pericytes [90,91]. Tsakogiannis and Wang et al. demonstrated that ANGPT2 destabilizes existing vessels, facilitating VEGF-induced neovascularization [92,93]. Raica and Cimpean demonstrated that PDGF-B supports pericyte recruitment and vessel maturation, improving stability and perfusion [94]. 

Besides the induction of pro-angiogenic factors, ECM remodeling and endothelial cell survival are also involved. For example, Gonzalez-Avila et al. demonstrated that HIF-1α upregulates matrix metalloproteinases (MMP-2, MMP-9), enabling endothelial invasion into hypoxic zones [95]. Ni et al. demonstrated that HIF-1α induces anti-apoptotic pathways in endothelial cells, protecting them from oxidative stress during neovascularization [96].

### 4.4. The Role of HIF-1α in Diabetic Testicular Cell Apoptosis

**Figure 12:**
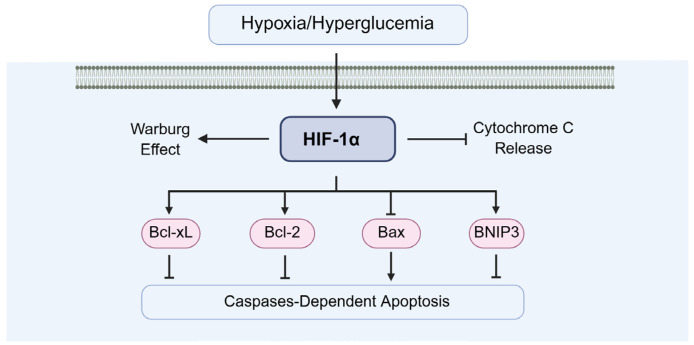
The of HIF-1α in diabetic testicular cell apoptosis. Bcl-2: B-cell lymphoma 2, Bax: Bcl-2 associated X.

In diabetes-induced testicular cell apoptosis, HIF-1α plays a dual role by facilitating cellular adaptation to hypoxia and protecting cells from apoptosis (Figure 12). One is the early-stage protective role of HIF-1α, including inducing anti-apoptotic proteins (Bcl-2, Bcl-xL), supporting glycolysis to maintain ATP production, boosting antioxidant defenses (HO-1, SOD2), and enhancing VEGF-mediated microvascular repair. For example, Li and Dong et al. demonstrated that HIF-1α exerts anti-apoptotic effects through modulation of Bcl-2 family proteins, suppressing Bax expression [16] while upregulating Bcl-2 [97]. Additionally, Li et al. reported that HIF-1α protects testicular cells by preventing the loss of mitochondrial membrane potential [98] and inhibiting cytochrome C release [85,99], thereby reducing caspase-3 activation and downstream apoptotic signaling. 

The other is the chronic-stage pro-apoptotic shift in HIF-1α, including the upregulation of p53 and Bax, the overactivation of BNIP3/NIX, the suppression of steroidogenesis, induction of inflammatory cytokines (IL-1β, IL-6, TNF-α), cytochrome c release, and caspase-9/-3 activation. For example, Piret et al. demonstrated that chronic HIF-1α upregulation can increase transcription of pro-apoptotic genes, promoting mitochondrial outer membrane permeabilization (MOMP) [100], and then MOMP releases cytochrome c, leading to caspase-9 and caspase-3 activation in germ cells [100]. Ma and Rambold et al. demonstrated that excessive mitophagy can deplete healthy mitochondria, impairing energy supply and triggering intrinsic apoptosis [101,102]. Palladino et al. demonstrated that HIF-1α upregulates IL-1β, IL-6, and TNF-α, which activate death receptor pathways (extrinsic apoptosis) in Sertoli cells [103].

In addition, ROS–HIF-1α feedback loop exists in diabetic testicular cells. ROS stabilizes HIF-1α, which in chronic conditions amplifies inflammatory and oxidative injury, perpetuating apoptotic signaling.

### 4.5. The Role of HIF-1α in Testicular Cell Functions

The role of HIF-1α in different testicular cells of diabetic testes, such as Leydig cells, Sertoli cells, spermatogonia, and spermatozoa, is reviewed as following.

**Figure 13:**
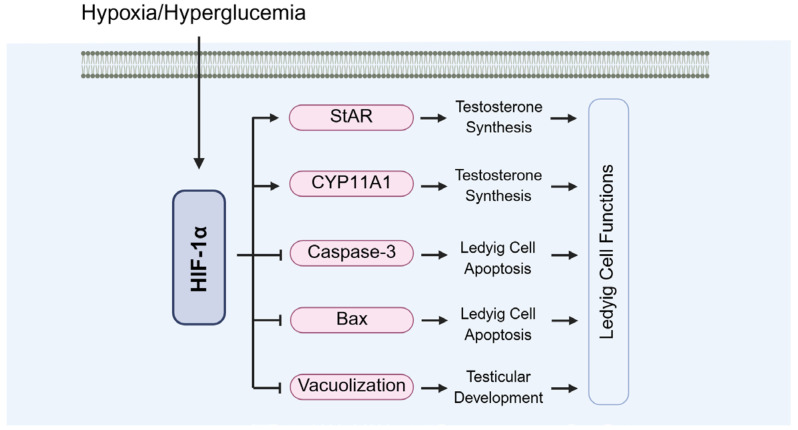
The role of HIF-1α in the functions of Leydig cells. StAR: steroidogenic acute regulatory protein; CYP11A1: cytochrome P450 family 11 subfamily A member 1.

Diabetes can impair Leydig cell function, leading to insufficient androgen secretion (Figure 13). Under hypoxic conditions, HIF-1α regulates the expressions of steroidogenesis-related genes, such as StAR and CYP11A1, thereby supporting Leydig cell synthetic capacity (Figure 13). Sui et al. demonstrated that loss of HIF-1α suppresses StAR expression, a critical regulator of testosterone biosynthesis [67]. Additionally, Hwang et al. found that HIF-1α influences testosterone production by modulating hypoxia-responsive pathways and oxidative stress [104].

In the diabetic testis, hyperglycemia-induced oxidative stress and microvascular dysfunction can lead to BTB disruption and germ cell loss. HIF-1α activation in Sertoli cells under these conditions appears to have a dual role. On one hand, HIF-1α promotes cytoprotective pathways, including antioxidant enzyme upregulation and autophagy, which mitigate ROS accumulation and preserve cellular homeostasis [105,106]. On the other hand, chronic hyperactivation or maladaptive HIF-1α signaling may exacerbate Sertoli cell dysfunction [105].

During diabetes-induced testicular injury, chronic hyperglycemia exacerbates mitochondrial dysfunction and oxidative damage in spermatogonial cells. HIF-1α can induce HO-1 and SOD expression, mitigating ROS accumulation to protect spermatogonia [107]. But chronic HIF-1α activation has been linked to the induction of pro-apoptotic factors such as BNIP3 and NIX, regulating spermatogonial apoptosis by the Bcl-2/Bax axis [108].

Mature spermatozoa are terminally differentiated cells with limited transcriptional capacity. However, HIF-1α plays important indirect and residual roles impacting sperm function and viability. Though sperm themselves have minimal active gene transcription, HIF-1α-mediated pathways in precursor cells and the testicular microenvironment critically influence sperm quality, motility, and survival [10,109,110]. In addition, elevated testicular or seminal oxidative stress and altered HIF-1α signaling have been correlated with reduced sperm motility, increased DNA fragmentation, and poor fertilization outcomes in diabetic and varicocele patients [105,110,111]. 

HIF-1α-deficient mice exhibit reduced testosterone levels and impaired testicular development (Figure 13). Zheng et al. reported that testes from HIF-1α-deficient mice showed damage to seminiferous tubules, vacuolation of Leydig cells, and a significant decline in sperm quality. These structural abnormalities further exacerbate disruptions in testosterone synthesis [112]. Moreover, Xu et al. found that HIF-1α deficiency increases testicular cell apoptosis through upregulating caspase-3 and Bax expressions [28].

Interestingly, HIF-1α plays distinct roles in different diabetic target organs. In the retina and kidneys, persistent HIF-1α activation under chronic hyperglycemia promotes pathological angiogenesis and fibrosis, contributing to diabetic retinopathy and nephropathy [113,114]. In contrast, within testicular tissue, where hypoxia naturally exists due to a specialized vascular barrier, moderate HIF-1α activity may be more beneficial—supporting spermatogenic cell metabolism and vascular integrity [105,106,107]. These organ-specific differences underscore the complexity of HIF-1α signaling and the need for tissue-targeted therapeutic strategies.

Additionally, conflicting evidence exists regarding the role of HIF-1α in male fertility. While several studies demonstrate protective effects of HIF-1α activation in promoting testicular angiogenesis and energy homeostasis [97,98,99], others report that sustained HIF-1α overexpression may exacerbate inflammation and apoptosis under diabetic conditions [100,101,102,103]. These discrepancies may stem from variations in experimental models, HIF-1α expression levels, or differences in species and disease progression. Therefore, the functional outcome of HIF-1α modulation appears to be dose-, duration-, and context-dependent.

## 5. The Signaling Pathways of HIF-1α Regulating Diabetic Testicular Injury

**Figure 14:**
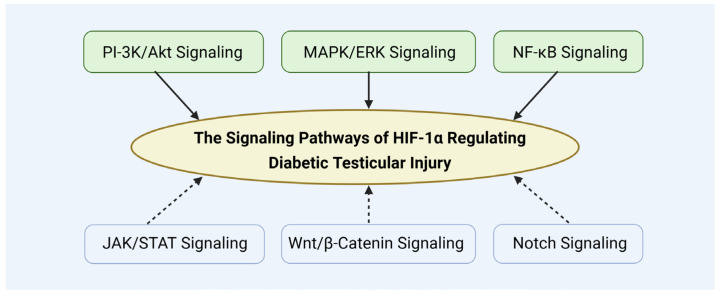
The signaling pathways of HIF-1α regulating diabetic testicular injury.

The upstream signaling pathways regulating HIF-1α are complex and multifaceted, with various mechanisms jointly modulating its expression and activity. Understanding these pathways offers critical insights into the pathogenesis of diabetes-induced testicular damage and establishes a theoretical foundation for targeting HIF-1α as a potential therapeutic strategy (Figure 14). 

### 5.1. PI-3K/Akt Signaling in Diabetic Testicular Injury

PI-3K/Akt signaling is pivotal during cell growth, proliferation, and survival. It can directly or indirectly modulate HIF-1α expression and activity, thereby enhancing cell survival and inhibiting apoptosis. Ma et al. demonstrated that PI-3K/Akt activation in a myocardial hypoxia/reoxygenation injury model significantly increased HIF-1α protein levels, helping to maintain mitochondrial dynamics and reduce apoptosis [115]. Zhang et al. found that NF90/ILF3 upregulated HIF-1α and VEGF expression via the PI-3K/Akt pathway, promoting angiogenesis and cell survival [116]. Additionally, Zheng et al. showed that parkinson disease protein 7 (PARK7/DJ-1) enhances cell survival and inhibits hypoxia-induced apoptosis through the PI-3K/Akt–HIF-1α axis [117].

In hypoxic conditions, PI-3K/Akt activation promotes the stabilization of HIF-1α, thereby enhancing its transcriptional activity and initiating the expression of multiple cytoprotective genes. An et al. demonstrated that PI-3K/Akt activation inhibits HIF-1α degradation, leading to its increased cellular accumulation [118]. Patra et al. further found that this pathway enhances HIF-1α transcriptional activity by regulating its translation and post-transcriptional modifications [119]. Collectively, the protective role of PI-3K/Akt-mediated HIF-1α regulation was highlighted in diabetes-induced injury, positioning this pathway as a promising therapeutic target.

### 5.2. MAPK/ERK Signaling in Diabetic Testicular Injury

MEK/ERK signaling is another critical upstream regulator of cell proliferation and survival. Activation of this pathway can enhance both the expression and activity of HIF-1α, contributing to the repair of diabetic testicular tissue. Haque et al. demonstrated that under hyperglycemic conditions, MEK/ERK activation promotes VEGF expression by increasing HIF-1α translocational activity, thereby facilitating angiogenesis and tissue repair [120]. Additionally, Zhang et al. found that 20(S)-Protopanaxadiol significantly accelerated wound healing in diabetic mice through activation of both the MEK/ERK and PI-3K/Akt/mTOR pathways, leading to enhanced HIF-1α-mediated VEGF expression [121].

In addition, MEK/ERK signaling modulates cell proliferation, differentiation, and inflammatory responses, thereby positively influencing the repair of diabetes-related testicular tissue damage. Liu et al. demonstrated that MEK/ERK activation enhances cell proliferation while inhibiting apoptosis, contributing to tissue repair [122]. Lin et al. found that this pathway suppresses proinflammatory cytokine release, like IL-6 and TNF-α, reducing inflammation-induced testicular injury [123]. Moreover, Qi et al. reported that MEK/ERK signaling regulates OS by decreasing ROS production, providing further protection against diabetes-induced oxidative damage to testicular tissue [124].

### 5.3. NF-κB Signaling in Diabetic Testicular Injury

NF-κB signaling is a canonical regulator of inflammation and immune responses, exhibiting a positive regulatory interplay with HIF-1α. Zhang et al. demonstrated that NF-κB directly binds to HIF-1α promoter, enhancing its transcription and thus increasing HIF-1α expression [125]. Wang et al. found that HIF-1α can further activate NLRP3 inflammasomes via the NF-κB signaling pathway, amplifying inflammatory responses [126]. Cheng and Lin et al. reported that HIF-1α cooperates with NF-κB to upregulate proinflammatory cytokines (e.g., IL-6 and TNF-α) [127,128], while also modulating inflammation through negative feedback mechanisms to prevent excessive inflammatory activation and tissue damage [129].

In addition, HIF-1α expression is also regulated by other signaling, including JAK/STAT pathway [130], Wnt/β-catenin pathway [131], and Notch pathway [132]. These pathways may also contribute to modulating HIF-1α’s role in diabetic testicular damage.

## 6. Potential Therapeutic Targets and Clinical Applications of HIF-1α

**Figure 15:**
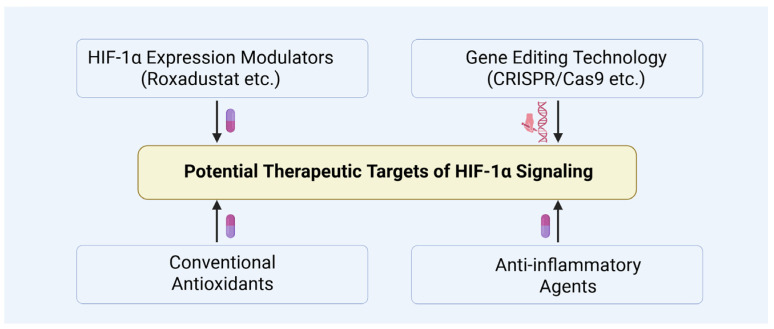
Potential therapeutic targets of HIF-1α signaling.

Several drugs capable of indirectly modulating HIF-1α expression, including PHD inhibitors such as Roxadustat and other oxygen-sensing signal modulators, have shown promise in treating hypoxia-related diseases (Figure 15) [83,133,134]. Sugahara et al. demonstrated that the PHD inhibitor enarodustat attenuates glomerular injury by activating the HIF pathway and inhibiting macrophage infiltration through downregulation of inflammatory mediators CCL2/MCP-1, thereby exerting protective effects against diabetic nephropathy [135]. Additionally, Miyata et al. reported that PHD inhibitors may have therapeutic potential for metabolic disorder-related diseases by regulating lipid metabolism and enhancing insulin sensitivity [136,137].

CRISPR/Cas9 may offer a promising avenue for precisely regulating HIF-1α expression, potentially enabling targeted interventions in diabetic testicular damage (Figure 15). For instance, HIF-1α inhibitors like PX-478 have demonstrated improvements in β-cell function within diabetic models, underscoring the therapeutic potential of modulating HIF-1α in diabetes and its complications [58]. Despite the significant promise of CRISPR/Cas9 for controlling HIF-1α expression, challenges related to its safety and efficacy remain, including the need to minimize off-target effects and enhance editing efficiency [138]. Furthermore, advancements in nanodelivery systems and localized application techniques may further facilitate precise therapeutic targeting of diabetic testicular injury [139].

Furthermore, combining HIF-1α-targeted therapies with conventional antioxidants and anti-inflammatory agents may yield synergistic benefits (Figure 15). For example, co-administration of n-acetylcysteine or curcumin with HIF-1α stabilizers has been shown to more effectively mitigate testicular tissue damage, highlighting the potential of such combination strategies for further investigation [140,141,142]. 

While HIF-1α modulators such as Roxadustat and Vadadustat are being tested for anemia and kidney-related complications in diabetes, their application in reproductive disorders remains limited. Preclinical studies using PHD inhibitors suggest potential benefits in testicular ischemia or diabetic infertility models. However, clinical trials in male reproductive health are still lacking. Further research is warranted to explore HIF-1α-targeted therapies in this context.

Additionally, there are animal studies of diabetic testicular dysfunction, apoptosis, and inflammation reporting improvements of oxidative stress after common antidiabetic drugs (metformin, GLP1RA, etc.). Inceu et al. demonstrated that GLP-1R activation can modulate HIF-1α stability and transcriptional activity via PI-3K/Akt and ERK1/2 signaling, promoting angiogenic and cytoprotective gene expression under metabolic stress [143]. Metformin not only promotes the moderate HIF-1α activation in Sertoli and germ cells to sustain VEGF, GLUT1, and EPO expressions, supporting nutrient supply and oxygen delivery [105,144], but also activates AMP-activated protein kinase, which can indirectly suppress excessive HIF-1α accumulation under hyperglycemic and inflammatory conditions by inhibiting mTOR signaling [145]. Thus, the effects of metformin on HIF-1α in the diabetic testis appear context-dependent, aiming to restore physiological hypoxic adaptation while suppressing hyperglycemia-induced maladaptive activation. 

## 7. Conclusions

HIF-1α plays a central and multifaceted regulatory role in diabetes-induced testicular injury, encompassing various pathological processes such as oxidative stress, local hypoxia, impaired angiogenesis, testicular cell apoptosis, autophagy imbalance, and endocrine dysfunction. As an oxygen-sensitive transcription factor, HIF-1α not only mitigates ROS-mediated oxidative damage by regulating antioxidant enzymes (e.g., SOD and GPx) but also enhances local testicular perfusion by upregulating angiogenic factors such as VEGF, thereby alleviating hypoxic stress. Its modulation of the Bcl-2/Bax axis further contributes to the regulation of spermatogonial apoptosis. Additionally, HIF-1α may be implicated in the pathogenesis of androgen synthesis disorders through its effects on Leydig cell function.

More importantly, HIF-1α interacts synergistically with multiple inflammatory mediators and metabolic regulators via several signaling pathways, including PI-3K/Akt, MAPK/ERK, and NF-κB, forming a highly intricate and dynamically regulated network. This positions HIF-1α not only as a “sensor” of disease progression, but also as a potential “modulator” for therapeutic intervention. Notably, the effects of HIF-1α exhibit both dose-dependence and time-window specificity; it may exert protective roles in the early phases of pathology but could contribute to proinflammatory or pro-fibrotic processes under prolonged pathological conditions.

Additionally, the limitations are that most mechanistic insights regarding HIF-1α in diabetic testicular injury are derived from rodent models (mice, rats), which may not fully recapitulate the complex endocrine and testicular architecture of humans. Species-specific differences in spermatogenesis, Sertoli cell function, and hypoxia responsiveness may limit direct clinical translation, emphasizing the need for confirmatory studies in human tissues or primate models.

In conclusion, as a pivotal transcription factor, the precise mechanisms by which HIF-1α mediates diabetic testicular injury remain to be fully elucidated. Future research focusing on the mechanistic pathways and translational potential of HIF-1α is expected to provide novel theoretical insights and therapeutic targets for the management of diabetes-related male infertility.

## Data Availability

Not applicable.

## Abbreviations

3β-Hsd2	3β-hydroxysteroid dehydrogenase 2
Akt/PKB	protein kinase B
bHLH	basic helix-loop-helix
BNIP3	B-cell leukemia/lymphoma 2/adenovirus E1B interacting protein 3
cAMP	cyclic adenosine monophosphate
CAT	catalase
CBP	CREB-binding protein
CCL2	chemokine (C-C motif) ligand 2
Cyp11A1	cytochrome P450 family 11 subfamily A polypeptide 1
EPO	erythropoietin
ERK	extracellular signal-regulated kinase
FSH	follicle-stimulating hormone
GLUT-1	glucose transporter-1
GPx	glutathione peroxidase
HIF-1α	hypoxia-inducible factor-1α
HPG	hypothalamic-pituitary-gonadal axis
HRE	hypoxia response element
IL-6	interleukin-6
LC-3	microtubule-associated proteins light chain 3
LDHA	lactate dehydrogenase A
LH	luteinizing hormone
MAPK	mitogen-activated protein kinase
MCP-1	monocyte chemoattractant protein 1
mTOR	mammalian target of rapamycin
NF90/ILF3	interleukin enhancer binding factor 3
NF-κB	nuclear factor-κB
NIX	Nip-like protein X
Nrf2	nuclear factor erythroid 2-related factor 2
OS	oxidative stress
p300	E1A binding protein p300
PARK7/DJ-1	Parkinson disease protein 7
PAS	Per-ARNT-Sim domain
PHD	prolyl hydroxylase
PI-3K	phosphoinositide-3 kinase
PKA	protein kinase A
ROS	reactive oxygen species
SOD	superoxide dismutase
StAR	steroidogenic acute regulatory protein
TNF-α	tumor necrosis factor-α
UCP2	uncoupling protein 2
VEGF	vascular endothelial growth factor
VHL	the von Hippel–Lindau protein

## References

[1] Chaurasia P.P., Dholariya S., Kotadiya F., Bhavsar M. (2021). A new hope in type 2 diabetes mellitus management: Sodium-glucose cotransporter 2 inhibitors. Cureus.

[2] Alam U., Asghar O., Azmi S., Malik R.A. (2014). General aspects of diabetes mellitus. Handb. Clin. Neurol..

[3] Maresch C.C., Stute D.C., Ludlow H., Hammes H.P., de Kretser D.M., Hedger M.P., Linn T. (2017). Hyperglycemia is associated with reduced testicular function and activin dysregulation in the *Ins2*^Akita+/−^ mouse model of type 1 diabetes. Mol. Cell Endocrinol..

[4] He Z., Yin G., Li Q.Q., Zeng Q., Duan J. (2021). Diabetes mellitus causes male reproductive dysfunction: A review of the evidence and mechanisms. In Vivo.

[5] Ding G.L., Liu Y., Liu M.E., Pan J.X., Guo M.X., Sheng J.Z., Huang H.F. (2015). The effects of diabetes on male fertility and epigenetic regulation during spermatogenesis. Asian J. Androl..

[6] Alyürük B., Yazir Y., Utkan Korun Z.E., Budak Ö., Yalçinkaya Kalyan E., Kiliç K.C. (2025). Impacts of type 1 diabetes mellitus on male fertility and embryo quality in superovulated mice. Tissue Cell.

[7] Musa E., El-Bashir J.M., Sani-Bello F., Bakari A.G. (2021). Clinical and biochemical correlates of hypogonadism in men with type 2 diabetes mellitus. Pan Afr. Med. J..

[8] Lotti F., Maggi M. (2023). Effects of diabetes mellitus on sperm quality and fertility outcomes: Clinical evidence. Andrology.

[9] Bi J., Zhou W., Tang Z. (2024). Pathogenesis of diabetic complications: Exploring hypoxic niche formation and HIF-1α activation. Biomed. Pharmacother..

[10] Xu R., Wang F., Zhang Z., Zhang Y., Tang Y., Bi J., Shi C., Wang D., Yang H., Wang Z. (2023). Diabetes-induced autophagy dysregulation engenders testicular impairment via oxidative stress. Oxid. Med. Cell Longev..

[11] Liu C., Cheng T., Wang Y., Li G., Wang Y., Tian W., Feng L., Zhang S., Xu Y., Gao Y. (2024). Syringaresinol alleviates early diabetic retinopathy by downregulating HIF-1α/VEGF via activating Nrf2 antioxidant pathway. Mol. Nutr. Food Res..

[12] Takahashi N., Davy P.M., Gardner L.H., Mathews J., Yamazaki Y., Allsopp R.C. (2016). Hypoxia inducible factor 1 alpha is expressed in germ cells throughout the murine life cycle. PLoS ONE.

[13] Powell J.D., Elshtein R., Forest D.J., Palladino M.A. (2002). Stimulation of hypoxia-inducible factor-1 alpha (HIF-1alpha) protein in the adult rat testis following ischemic injury occurs without an increase in HIF-1alpha messenger RNA expression. Biol. Reprod..

[14] Wang P., Zhang X.P., Liu F., Wang W. (2025). Progressive deactivation of hydroxylases controls hypoxia-inducible factor-1α-coordinated cellular adaptation to graded hypoxia. Research.

[15] Han Y., Koohi-Moghadam M., Chen Q., Zhang L., Chopra H., Zhang J., Dissanayaka W.L. (2022). HIF-1alpha stabilization boosts pulp regeneration by modulating cell metabolism. J. Dent. Res..

[16] Li S., Zhao J., Xi Y., Ren J., Zhu Y., Lu Y., Dong D. (2023). Dl-3-n-butylphthalide exerts neuroprotective effects by modulating hypoxia-inducible factor 1-alpha ubiquitination to attenuate oxidative stress-induced apoptosis. Neural Regen. Res..

[17] Wang D., Gao Z., Zhang X. (2018). Resveratrol induces apoptosis in murine prostate cancer cells via hypoxia-inducible factor 1-alpha (HIF-1α)/reactive oxygen species (ROS)/P53 signaling. Med. Sci. Monit..

[18] Zeng D., Zhou P., Jiang R., Li X.P., Huang S.Y., Li D.Y., Li G.L., Li L.S., Zhao S., Hu L. (2021). Evodiamine inhibits vasculogenic mimicry in HCT116 cells by suppressing hypoxia-inducible factor 1-alpha-mediated angiogenesis. Anticancer Drugs.

[19] Li A., Li M., Xu S., Niu L., Li S., Zhou Y., Cao Z., Cai R., He B., Guo A. (2025). HIF-1α-induced astrocytic D-dopachrome tautomerase activates microglial inflammatory response following spinal cord injury. Am. J. Pathol..

[20] Bai Z., Zhou D., Tao K., Lin F., Wang H., Sun H., Liu R., Li Z. (2025). The role of microRNA-206 in the regulation of diabetic wound healing via hypoxia-inducible factor 1-alpha. Biochem. Genet..

[21] Wang X., Liu M., Wu Y., Sun J., Liu L., Pan Z. (2025). Gentiopicroside targeting AKT1 activates HIF-1α/VEGF axis promoting diabetic ulcer wound healing. Front. Pharmacol..

[22] Lei J., Jiang X., Huang D., Jing Y., Yang S., Geng L., Yan Y., Zheng F., Cheng F., Zhang W. (2024). Human ESC-derived vascular cells promote vascular regeneration in a HIF-1α dependent manner. Protein Cell.

[23] Basheeruddin M., Qausain S. (2024). Hypoxia-inducible factor 1-alpha (HIF-1α): An essential regulator in cellular metabolic Control. Cureus.

[24] Yang F., Huang R., Ma H., Zhao X., Wang G. (2020). miRNA-411 regulates chondrocyte autophagy in osteoarthritis by targeting hypoxia-Inducible factor 1 alpha (HIF-1alpha). Med. Sci. Monit..

[25] Thangwong P., Tocharus C., Tocharus J. (2025). The bidirectional role of hypoxia-inducible factor 1 alpha in vascular dementia caused by chronic cerebral hypoperfusion. Mol. Neurobiol..

[26] Zhang Z., Wang D., Xu R., Li X., Wang Z., Zhang Y. (2024). The physiological functions and therapeutic potential of hypoxia-inducible factor-1α in vascular calcification. Biomolecules.

[27] Zhang Z., Yin D., Wang Z. (2011). Contribution of hypoxia-inducible factor-1α to transcriptional regulation of vascular endothelial growth factor in bovine developing luteal cells. Anim. Sci. J..

[28] Xu R., Shen S., Wang D., Ye J., Song S., Wang Z., Yue Z. (2023). The role of HIF-1alpha-mediated autophagy in ionizing radiation-induced testicular injury. J. Mol. Histol..

[29] Zhang Z., Shi C., Wang Z. (2023). Therapeutic effects and molecular mechanism of chlorogenic acid on polycystic ovarian syndrome: Role of HIF-1alpha. Nutrients.

[30] Shi C., Zhang Z., Xu R., Zhang Y., Wang Z. (2023). Contribution of HIF-1alpha/BNIP3-mediated autophagy to lipid accumulation during irinotecan-induced liver injury. Sci. Rep..

[31] Zhang H., Xu R., Wang Z. (2021). Contribution of oxidative stress to HIF-1-mediated profibrotic changes during the kidney damage. Oxid. Med. Cell Longev..

[32] Wang Z., Zhu Q., Li P.L., Dhaduk R., Zhang F., Gehr T.W., Li N. (2014). Silencing of hypoxia-inducible factor-1α gene attenuates chronic ischemic renal injury in two-kidney.; one-clip rats. Am. J. Physiol. Renal Physiol..

[33] Wang Z., Tang L., Zhu Q., Yi F., Zhang F., Li P.L., Li N. (2011). Hypoxia-inducible factor-1α contributes to the profibrotic action of angiotensin II in renal medullary interstitial cells. Kidney Int..

[34] Gong X., Yang S., Yuan Z., Zhang Z., Ali F., Zhang F., Zhang B. (2025). Diosmetin attenuates the ubiquitination of epidermal hypoxia-inducible factor 1 alpha by diminishing the formation of RhoBTB3/PHD2 complex in ultraviolet radiation-induced sunburn in mice. Phytomedicine.

[35] Kim H., Park C., Wei X., Chhetri A., Manandhar L., Jang G., Hwang J., Chinbold B., Chuluunbaatar C., Kwon H.M. (2025). Golgi condensation causes intestinal lipid accumulation through HIF-1α-mediated GM130 ubiquitination by NEDD4. Exp. Mol. Med..

[36] Wang Z., Zhu Q., Xia M., Li P.L., Hinton S.J., Li N. (2010). Hypoxia-inducible factor prolyl-hydroxylase 2 senses high-salt intake to increase hypoxia inducible factor 1alpha levels in the renal medulla. Hypertension.

[37] Yang Z., Su W., Wei X., Pan Y., Xing M., Niu L., Feng B., Kong W., Ren X., Huang F. (2025). Hypoxia inducible factor-1α drives cancer resistance to cuproptosis. Cancer Cell.

[38] Basheeruddin M., Qausain S. (2024). Hypoxia-inducible factor 1-alpha (HIF-1α) and cancer: Mechanisms of tumor hypoxia and therapeutic targeting. Cureus.

[39] Li Y., Zhu R., He X., Song Y., Fan T., Ma J., Xiang G., Ma X. (2024). Discovery of potent hypoxia-inducible factor-1α (HIF-1α) degraders by proteolysis targeting chimera (PROTAC). Bioorg. Chem..

[40] Meng K., Liu Q., Qin Y., Qin W., Zhu Z., Sun L., Jiang M., Adu-Amankwaah J., Gao F., Tan R. (2025). Mechanism of mitochondrial oxidative phosphorylation disorder in male infertility. Chin. Med. J..

[41] Zhang X., Tu H., Zhou X., Wang B., Guo Y., Situ C., Qi Y., Li Y., Guo X. (2024). Quantitative phosphoproteomic profiling of mouse sperm maturation in epididymis revealed kinases important for sperm motility. Mol. Cell. Proteom..

[42] Chang Z., Miao L., Wang P. (2025). Mitochondrial ribosome regulation drives spermatogenesis and male fertility. Biol. Cell.

[43] Miyabayashi K., Shima Y., Inoue M., Sato T., Baba T., Ohkawa Y., Suyama M., Morohashi K.I. (2017). Alterations in fetal leydig cell gene expression during fetal and adult development. Sex. Dev..

[44] Geetha A.V.S., Harithpriya K., Ganesan K., Ramkumar K.M. (2025). Exploring the role of hypoxia and HIF-1α in the intersection of type 2 diabetes mellitus and endometrial cancer. Curr. Oncol..

[45] Islam K., Islam R., Nguyen I., Malik H., Pirzadah H., Shrestha B., Lentz I.B., Shekoohi S., Kaye A.D. (2025). Diabetes mellitus and associated vascular disease: Pathogenesis, complications, and evolving treatments. Adv. Ther..

[46] Song X., Fan C., Wei C., Yu W., Tang J., Ma F., Chen Y., Wu B. (2024). Mitochondria fission accentuates oxidative stress in hyperglycemia-induced H9c2 cardiomyoblasts in vitro by regulating fatty acid oxidation. Cell Biol. Int..

[47] Minas A., Camargo M., Alves M.G., Bertolla R.P. (2024). Effects of diabetes-induced hyperglycemia on epigenetic modifications and DNA packaging and methylation during spermatogenesis; A narrative review. Iran. J. Basic. Med. Sci..

[48] Biasi A., Ambruosi M.R., Romano M.Z., Boccella S., Falvo S., Guida F., Aniello F., Maione S., Venditti M., Minucci S. (2025). Impact of type 1 diabetes on testicular microtubule mynamics, sperm physiology, and male reproductive health in rat. Int. J. Mol. Sci..

[49] Kilarkaje N., Al-Hussaini H., Al-Bader M.M. (2014). Diabetes-induced DNA damage and apoptosis are associated with poly (ADP ribose) polymerase 1 inhibition in the rat testis. Eur. J. Pharmacol..

[50] Iskender H., Dokumacioglu E., Sen T.M., Ince I., Kanbay Y., Saral S. (2017). The effect of hesperidin and quercetin on oxidative stress.; NF-κB and SIRT1 levels in a STZ-induced experimental diabetes model. Biomed. Pharmacother..

[51] Qiu D., Song S., Wang Y., Bian Y., Wu M., Wu H., Shi Y., Duan H. (2022). NAD(P)H: Quinone oxidoreductase 1 attenuates oxidative stress and apoptosis by regulating Sirt1 in diabetic nephropathy. J. Transl. Med..

[52] Yoshikawa T., Mifune Y., Inui A., Nishimoto H., Yamaura K., Mukohara S., Shinohara I., Kuroda R. (2022). Quercetin treatment protects the Achilles tendons of rats from oxidative stress induced by hyperglycemia. BMC Musculoskelet. Disord..

[53] Goligorsky M.S. (2023). Glomerular microcirculation: Implications for diabetes.; preeclampsia.; and kidney injury. Acta Physiol..

[54] Fagundes R.R., Zaldumbide A., Taylor C.T. (2024). Role of hypoxia-inducible factor 1 in type 1 diabetes. Trends Pharmacol. Sci..

[55] Alyami N.M., Alnakhli Z.A., Alshiban N.M., Maodaa S., Almuhaini G.A., Almeer R., Alshora D., Ibrahim M. (2024). Oral administration of proniosomal glibenclamide formulation protects testicular tissue from hyperglycemia fluctuations and ROS via Nrf2/HO-1 pathway. Heliyon.

[56] Bowker R.M., Yan X., De Plaen I.G. (2018). Intestinal microcirculation and necrotizing enterocolitis: The vascular endothelial growth factor system. Semin. Fetal Neonatal Med..

[57] Long L., Qiu H., Cai B., Chen N., Lu X., Zheng S., Ye X., Li Y. (2018). Hyperglycemia induced testicular damage in type 2 diabetes mellitus rats exhibiting microcirculation impairments associated with vascular endothelial growth factor decreased via PI3K/Akt pathway. Oncotarget.

[58] Ilegems E., Bryzgalova G., Correia J., Yesildag B., Berra E., Ruas J.L., Pereira T.S., Berggren P.O. (2022). HIF-1a inhibitor PX-478 preserves pancreatic b cell function in diabetes. Sci. Transl. Med..

[59] Zhuang S., Sun N., Qu J., Chen Q., Han C., Yin H., Yuan X., Zhang M. (2025). High glucose/ChREBP-induced Hif-1α transcriptional activation in CD4+ T cells reduces the risk of diabetic kidney disease by inhibiting the Th1 response. Diabetologia.

[60] Graziani A., Scafa R., Grande G., Ferlin A. (2024). Diabetes and male fertility disorders. Mol. Asp. Med..

[61] Facondo P., Di Lodovico E., Delbarba A., Anelli V., Pezzaioli L.C., Filippini E., Cappelli C., Corona G., Ferlin A. (2022). The impact of diabetes mellitus type 1 on male fertility: Systematic review and meta-analysis. Andrology.

[62] Pelusi C. (2022). The effects of the new therapeutic treatments for diabetes mellitus on the male reproductive axis. Front. Endocrinol..

[63] Abd El-Twab S.M., Mohamed H.M., Mahmoud A.M. (2016). Taurine and pioglitazone attenuate diabetes-induced testicular damage by abrogation of oxidative stress and up-regulation of the pituitary-gonadal axis. Can. J. Physiol. Pharmacol..

[64] Wagner I.V., Klöting N., Savchuk I., Eifler L., Kulle A., Kralisch-Jäcklein S., Dötsch J., Hiort O., Svechnikov K., Söder O. (2021). Diabetes type 1 negatively influences leydig cell function in rats, which is partially reversible by insulin treatment. Endocrinology.

[65] Mo J.Y., Yan Y.S., Lin Z.L., Liu R., Liu X.Q., Wu H.Y., Yu J.E., Huang Y.T., Sheng J.Z., Huang H.F. (2022). Gestational diabetes mellitus suppresses fetal testis development in mice. Biol. Reprod..

[66] Dhole B., Gupta S., Kumar A. (2021). Triiodothyronine stimulates steroid and VEGF production in murine Leydig cells via cAMP-PKA pathway. Andrologia.

[67] Sui A., Yao C., Chen Y., Li Y., Yu S., Qu J., Wei H., Tang J., Chen G. (2023). Polystyrene nanoplastics inhibit StAR expression by activating HIF-1α via ERK1/2 MAPK and AKT pathways in TM3 Leydig cells and testicular tissues of mice. Food Chem. Toxicol..

[68] Buhur A., Gürel Ç., Kuşçu G.C., Yiğittürk G., Oltulu F., Karabay Yavaşoğlu N.Ü., Uysal A., Yavaşoğlu A. (2023). Is losartan a promising agent for the treatment of type 1 diabetes-induced testicular germ cell apoptosis in rats?. Mol. Biol. Rep..

[69] Du Z., Qiu Z., Wang Z., Wang X. (2018). The inhibitory effects of soybean isoflavones on testicular cell apoptosis in mice with type 2 diabetes. Exp. Ther. Med..

[70] Shoorei H., Khaki A., Khaki A.A., Hemmati A.A., Moghimian M., Shokoohi M. (2019). The ameliorative effect of carvacrol on oxidative stress and germ cell apoptosis in testicular tissue of adult diabetic rats. Biomed. Pharmacother..

[71] Tian Y., Xiao Y.H., Geng T., Sun C., Gu J., Tang K.F., Liu B., Liu Y.M., Sun F. (2021). Clusterin suppresses spermatogenic cell apoptosis to alleviate diabetes-induced testicular damage by inhibiting autophagy via the PI3K/AKT/mTOR axis. Biol. Cell.

[72] Tvrdá E., Kováč J., Benko F., Ďuračka M., Varga A., Uličná O., Almášiová V., Capcarová M., Chomová M. (2022). Characterization of the structural.; oxidative.; and immunological features of testis tissue from Zucker diabetic fatty rats. Open Life Sci..

[73] Naderi R., Pourheydar B., Moslehi A. (2023). Tropisetron improved testicular inflammation in the streptozotocin-induced diabetic rats: The role of toll-like receptor 4 (TLR4) and mir146a. J. Biochem. Mol. Toxicol..

[74] Wang X., Wei L., Li Q., Lai Y. (2022). HIF-1α protects osteoblasts from ROS-induced apoptosis. Free Radic. Res..

[75] Pan Y., Wu G., Chen M., Lu X., Shen M., Li H., Liu H. (2024). Lactate promotes hypoxic granulosa cells’ autophagy by activating the HIF-1α/BNIP3/Beclin-1 signaling axis. Genes.

[76] Abo El-Ella D.M. (2022). Autophagy/apoptosis induced by geraniol through HIF-1α/BNIP_3_/Beclin-1 signaling pathway in A_549_ CoCl_2_ treated cells. Adv. Pharm. Bull..

[77] Xiong S., Liu Z., Yao J., Huang S., Ding X., Yu H., Lin T., Zhang X., Zhao F. (2025). HIF-1α regulated GLUT1-mediated glycolysis enhances Treponema pallidum-induced cytokine responses. Cell Commun. Signal..

[78] Liu J., Hu X., Feng L., Lin Y., Liang S., Zhu Z., Shi S., Dong C. (2022). Carbonic anhydrase IX-targeted H-APBC nanosystem combined with phototherapy facilitates the efficacy of PI3K/mTOR inhibitor and resists HIF-1α-dependent tumor hypoxia adaptation. J. Nanobiotechnol..

[79] Baset M.A., El Awdan S.A., Khattab M.S., El-Marasy S.A. (2025). Involvement of hypoxia-inducible factor1-alpha in the protective effect of rivaroxaban against testicular ischemia-reperfusion in rats. Sci. Rep..

[80] Wang D., Zhao W., Liu J., Wang Y., Yuan C., Zhang F., Jin G., Qin Q. (2021). Effects of HIF-1alpha on spermatogenesis of varicocele rats by regulating VEGF/PI3K/Akt signaling pathway. Reprod. Sci..

[81] Lui W.Y., Cheng C.Y. (2007). Regulation of cell junction dynamics by cytokines in the testis: A molecular and biochemical perspective. Cytokine Growth Factor Rev..

[82] Lui W.Y., Wong E.W., Guan Y., Lee W.M. (2007). Dual transcriptional control of claudin-11 via an overlapping GATA/NF-Y motif: Positive regulation through the interaction of GATA, NF-YA, and CREB and negative regulation through the interaction of Smad, HDAC1, and mSin3A. J. Cell Physiol..

[83] Fang T., Ma C., Yang B., Zhao M., Sun L., Zheng N. (2025). Roxadustat improves diabetic myocardial injury by upregulating HIF-1α/UCP2 against oxidative stress. Cardiovasc. Diabetol..

[84] Hou Z., Wang Z., Zhang J., Liu Y., Luo Z. (2024). Effects of cannabidiol on AMPKalpha2 /HIF-1alpha/BNIP3/NIX signaling pathway in skeletal muscle injury. Front. Pharmacol..

[85] Wu J.J., Zhang S.Y., Mu L., Dong Z.G., Zhang Y.J. (2024). Heyingwuzi formulation alleviates diabetic retinopathy by promoting mitophagy via the HIF-1α/BNIP3/NIX axis. World J. Diabetes.

[86] Michalak K.P., Michalak A.Z. (2025). Understanding chronic inflammation: Couplings between cytokines, ROS, NO, Ca_i_^2+^, HIF-1α, Nrf2 and autophagy. Front. Immunol..

[87] Wang K., Dai X., He J., Yan X., Yang C., Fan X., Sun S., Chen J., Xu J., Deng Z. (2020). Endothelial overexpression of metallothionein prevents diabetes-induced impairment in ischemia angiogenesis through preservation of HIF-1α/SDF-1/VEGF signaling in endothelial progenitor cells. Diabetes.

[88] Chen Y., Yin W., Liu Z., Lu G., Zhang X., Yang J., Huang Y., Hu X., Chen C., Shang R. (2025). Exosomes derived from fibroblasts enhance skin wound angiogenesis by regulating HIF-1α/VEGF/VEGFR pathway. Burn. Trauma.

[89] Huang X., Zheng L., Zhou Y., Hu S., Ning W., Li S., Lin Z., Huang S. (2024). Controllable adaptive molybdate-oligosaccharide nanoparticles regulate M2 macrophage mitochondrial function and promote angiogenesis via PI3K/HIF-1α/VEGF pathway to accelerate diabetic wound healing. Adv. Healthc. Mater..

[90] Oda H., Nagamatsu T., Cabral H., Miyazaki T., Iriyama T., Kawana K., Fujii T., Osuga Y. (2021). Thrombomodulin promotes placental function by up-regulating placental growth factor via inhibition of high-mobility-group box 1 and hypoxia-inducible factor 1α. Placenta.

[91] Hsu H.W., Wall N.R., Hsueh C.T., Kim S., Ferris R.L., Chen C.S., Mirshahidi S. (2014). Combination antiangiogenic therapy and radiation in head and neck cancers. Oral Oncol..

[92] Tsakogiannis D., Nikolakopoulou A., Zagouri F., Stratakos G., Syrigos K., Zografos E., Koulouris N., Bletsa G. (2021). Update overview of the role of angiopoietins in lung cancer. Medicina.

[93] Wang Q., Lash G.E. (2017). Angiopoietin 2 in placentation and tumor biology: The yin and yang of vascular biology. Placenta.

[94] Raica M., Cimpean A.M. (2010). Platelet-derived growth factor (PDGF)/PDGF receptors (PDGFR) axis as target for antitumor and antiangiogenic therapy. Pharmaceuticals.

[95] Gonzalez-Avila G., Sommer B., Flores-Soto E., Aquino-Galvez A. (2023). Hypoxic effects on matrix metalloproteinases’ expression in the tumor microenvironment and therapeutic perspectives. Int. J. Mol. Sci..

[96] Ni J., Zhang Q., Jiang L., Wang H., Zhang C., Deng J. (2024). Catalpol regulates apoptosis and proliferation of endothelial cell via activating HIF-1α/VEGF signaling pathway. Sci. Rep..

[97] Dong J., Xu M., Zhang W., Che X. (2019). Effects of sevoflurane pretreatment on myocardial ischemia-reperfusion injury through the Akt/hypoxia-inducible factor 1-alpha (HIF-1α)/vascular endothelial growth factor (VEGF) signaling pathway. Med. Sci. Monit..

[98] Li H.S., Zhou Y.N., Li L., Li S.F., Long D., Chen X.L., Zhang J.B., Feng L., Li Y.P. (2019). HIF-1α protects against oxidative stress by directly targeting mitochondria. Redox Biol..

[99] Chen Z., Liu T., Xiong L., Liu Z. (2025). Shen-fu Injection modulates HIF- 1α/BNIP3-mediated mitophagy to alleviate myocardial ischemia-reperfusion injury. Cardiovasc. Toxicol..

[100] Piret J.P., Mottet D., Raes M., Michiels C. (2002). Is HIF-1alpha a pro- or an anti-apoptotic protein?. Biochem. Pharmacol..

[101] Ma K., Chen G., Li W., Kepp O., Zhu Y., Chen Q. (2020). Mitophagy, mitochondrial homeostasis, and cell fate. Front. Cell Dev. Biol..

[102] Rambold A.S., Lippincott-Schwartz J. (2011). Mechanisms of mitochondria and autophagy crosstalk. Cell Cycle.

[103] Palladino M.A., Fasano G.A., Patel D., Dugan C., London M. (2018). Effects of lipopolysaccharide-induced inflammation on hypoxia and inflammatory gene expression pathways of the rat testis. Basic Clin. Androl..

[104] Hwang T.I., Liao T.L., Lin J.F., Lin Y.C., Lee S.Y., Lai Y.C., Kao S.H. (2011). Low-dose testosterone treatment decreases oxidative damage in TM3 Leydig cells. Asian J. Androl..

[105] Chen Z.F., Shen Y.F., Gao D.W., Lin D.F., Ma W.Z., Chang D.G. (2025). Metabolic pathways and male fertility: Exploring the role of Sertoli cells in energy homeostasis and spermatogenesis. Am. J. Physiol. Endocrinol. Metab..

[106] Jiang Q., Di Q., Shan D., Xu Q. (2022). Nonylphenol inhibited HIF-1alpha regulated aerobic glycolysis and induced ROS mediated apoptosis in rat Sertoli cells. Ecotoxicol. Environ. Saf..

[107] Xiao S., Cui J., Cao Y., Zhang Y., Yang J., Zheng L., Zhao F., Liu X., Zhou Z., Liu D. (2024). Adolescent exposure to organophosphate insecticide malathion induces spermatogenesis dysfunction in mice by activating the HIF-1/MAPK/PI3K pathway. Environ. Pollut..

[108] Peng Y., Fang Z., Liu M., Wang Z., Li L., Ming S., Lu C., Dong H., Zhang W., Wang Q. (2019). Testosterone induces renal tubular epithelial cell death through the HIF-1alpha/BNIP3 pathway. J. Transl. Med..

[109] Hu J., Wu J., Liu X., Zhang Y., Mo L., Liu L., Liu S., Ou C., He Y. (2024). Hypoxia enhances autophagy level of human sperms. Sci. Rep..

[110] Li Z., Wang S., Gong C., Hu Y., Liu J., Wang W., Chen Y., Liao Q., He B., Huang Y. (2021). Effects of environmental and pathological hypoxia on male fertility. Front. Cell Dev. Biol..

[111] Babaei A., Moradi S., Hoseinkhani Z., Rezazadeh D., Dokaneheifard S., Asadpour R., Sharma G., Mansouri K. (2022). Expression of hypoxia-inducible factor1-alpha in varicocele disease: A comprehensive systematic review. Reprod. Sci..

[112] Zheng S., Jiang J., Shu Z., Qiu C., Jiang L., Zhao N., Lin X., Qian Y., Liang B., Qiu L. (2024). Fine particulate matter (PM2.5) induces testosterone disruption by triggering ferroptosis through SIRT1/HIF-1α signaling pathway in male mice. Free Radic. Biol. Med..

[113] Stanigut A.M., Pana C., Enciu M., Deacu M., Cimpineanu B., Tuta L.A. (2022). Hypoxia-inducible factors and diabetic kidney disease-how deep can we go?. Int. J. Mol. Sci..

[114] Min J., Zeng T., Roux M., Lazar D., Chen L., Tudzarova S. (2021). The role of HIF1α-PFKFB3 pathway in diabetic retinopathy. J. Clin. Endocrinol. Metab..

[115] Ma H., Hou T., Wu J., Zhao J., Cao H., Masula M., Wang J. (2024). Sevoflurane postconditioning attenuates cardiomyocytes hypoxia/reoxygenation injury via PI3K/AKT pathway mediated HIF-1α to regulate the mitochondrial dynamic balance. BMC Cardiovasc. Disord..

[116] Zhang W., Xiong Z., Wei T., Li Q., Tan Y., Ling L., Feng X. (2018). Nuclear factor 90 promotes angiogenesis by regulating HIF-1α/VEGF-A expression through the PI3K/Akt signaling pathway in human cervical cancer. Cell Death Dis..

[117] Zheng H., Zhou C., Lu X., Liu Q., Liu M., Chen G., Chen W., Wang S., Qiu Y. (2018). DJ-1 promotes survival of human colon cancer cells under hypoxia by modulating HIF-1α expression through the PI3K-AKT pathway. Cancer Manag. Res..

[118] An T.Y., Hu Q.M., Ni P., Hua Y.Q., Wang D., Duan G.C., Chen S.Y., Jia B. (2024). N6-methyladenosine modification of hypoxia-inducible factor-1α regulates Helicobacter pylori-associated gastric cancer via the PI3K/AKT pathway. World J. Gastrointest. Oncol..

[119] Patra K., Jana S., Sarkar A., Mandal D.P., Bhattacharjee S. (2019). The inhibition of hypoxia-induced angiogenesis and metastasis by cinnamaldehyde is mediated by decreasing HIF-1α protein synthesis via PI3K/Akt pathway. Biofactors.

[120] Haque R., Iuvone P.M., He L., Choi K.S.C., Ngo A., Gokhale S., Aseem M., Park D. (2017). The MicroRNA-21 signaling pathway is involved in prorenin receptor (PRR) -induced VEGF expression in ARPE-19 cells under a hyperglycemic condition. Mol. Vis..

[121] Zhang E.Y., Gao B., Shi H.L., Huang L.F., Yang L., Wu X.J., Wang Z.T. (2017). 20(S)-Protopanaxadiol enhances angiogenesis via HIF-1α-mediated VEGF secretion by activating p70S6 kinase and benefits wound healing in genetically diabetic mice. Exp. Mol. Med..

[122] Liu W., Li T., Hu W., Ji Q., Hu F., Wang Q., Yang X., Qi D., Chen H., Zhang X. (2021). Hematopoietic cell kinase enhances osteosarcoma development via the MEK/ERK pathway. J. Cell Mol. Med..

[123] Lin F., Zhou W., Yuan X., Liu S., He Z. (2024). Mechanistic study of quercetin in the treatment of hepatocellular carcinoma with diabetes via MEK/ERK pathway. Int. Immunopharmacol..

[124] Qi T., Qin H., Yu F., Zhou Z., Chen Y., Liu P., Zeng H., Weng J. (2025). XLOC_015548 mitigates skeletal muscle atrophy via the Gadd45g/MEK/ERK pathway and redox regulation. Front. Biosci.-Landmark.

[125] Zhang Z., Huang Y., Zhang J., Liu Z., Lin Q., Wang Z. (2019). Activation of NF-κB signaling pathway during HCG-induced VEGF expression in luteal cells. Cell Biol. Int..

[126] Wang D., Wang M., Sun S., Zhang C., Song Y., Li J., Song B., Lv H., Wang S., Jiang W. (2024). Hypoxia-induced NLRP3 inflammasome activation via the HIF-1α/NF-κB signaling pathway in human dental pulp fibroblasts. BMC Oral Health.

[127] Cheng J., Sun Y., He J., Wang Z., Li W., Wang R. (2022). The mechanism of colon tissue damage mediated by HIF-1α/NF-κB/STAT1 in high-altitude environment. Front. Physiol..

[128] Lin H., Chen M., Gao Y., Wang Z., Jin F. (2022). Tussilagone protects acute lung injury from PM2.5 via alleviating Hif-1α/NF-κB-mediated inflammatory response. Environ. Toxicol..

[129] Liu F.C., Yang Y.H., Liao C.C., Lee H.C. (2024). Xanthoxylin attenuates lipopolysaccharide-induced lung injury through modulation of Akt/HIF-1α/NF-κB and Nrf2 pathways. Int. J. Mol. Sci..

[130] Chen L., Zhou D., Liu Z., Huang X., Liu Q., Kang Y., Chen Z., Guo Y., Zhu H., Sun C. (2018). Combination of gemcitabine and erlotinib inhibits recurrent pancreatic cancer growth in mice via the JAK-STAT pathway. Oncol. Rep..

[131] Vallée A., Lecarpentier Y., Vallée J.N. (2021). The key role of the WNT/β-catenin pathway in metabolic reprogramming in cancers under normoxic conditions. Cancers.

[132] Qiang L., Wu T., Zhang H.W., Lu N., Hu R., Wang Y.J., Zhao L., Chen F.H., Wang X.T., You Q.D. (2012). HIF-1alpha is critical for hypoxia-mediated maintenance of glioblastoma stem cells by activating Notch signaling pathway. Cell Death Differ..

[133] Wang Y., Xiao M., Cai F., Li Y., Shi T., Zhou X., Tian S., Huang D. (2024). Roxadustat ameliorates vascular calcification in CKD rats by regulating HIF-2α/HIF-1α. Environ. Toxicol..

[134] Yang D.G., Gao Y.Y., Yin Z.Q., Wang X.R., Meng X.S., Zou T.F., Duan Y.J., Chen Y.L., Liao C.Z., Xie Z.L. (2023). Roxadustat alleviates nitroglycerin-induced migraine in mice by regulating HIF-1α/NF-κB/inflammation pathway. Acta Pharmacol. Sin..

[135] Sugahara M., Tanaka S., Tanaka T., Saito H., Ishimoto Y., Wakashima T., Ueda M., Fukui K., Shimizu A., Inagi R. (2020). Prolyl hydroxylase domain inhibitor protects against metabolic disorders and associated kidney disease in obese type 2 diabetic mice. J. Am. Soc. Nephrol..

[136] Miyata T., Suzuki N., van Ypersele de Strihou C. (2013). Diabetic nephropathy: Are there new and potentially promising therapies targeting oxygen biology?. Kidney Int..

[137] Miyata T., van Ypersele de Strihou C. (2010). Diabetic nephropathy: A disorder of oxygen metabolism?. Nat. Rev. Nephrol..

[138] Pandey S., Choudhari J.K., Tripathi A., Singh A., Antony A., Chouhan U. (2025). Artificial intelligence-based genome editing in CRISPR/Cas9. Methods Mol. Biol..

[139] Saber S., Abdelhady R., Elhemely M.A., Elmorsy E.A., Hamad R.S., Abdel-Reheim M.A., El-Kott A.F., AlShehri M.A., Morsy K., Negm S. (2024). Nanoscale systems for local activation of hypoxia-inducible factor-1 alpha: A new approach in diabetic wound management. Int. J. Nanomed..

[140] Shokoohi M., Khaki A.A., Roshangar L., Nasr Esfahani M.H., Soltani G.G., Alihemmati A. (2024). The impact of N-acetylcysteine on hypoxia-induced testicular apoptosis in male rats: TUNEL and IHC findings. Heliyon.

[141] Chowdhury R., Godoy L.C., Thiantanawat A., Trudel L.J., Deen W.M., Wogan G.N. (2012). Nitric oxide produced endogenously is responsible for hypoxia-induced HIF-1α stabilization in colon carcinoma cells. Chem. Res. Toxicol..

[142] Aktaş B.K., Bulut S., Bulut S., Baykam M.M., Ozden C., Senes M., Yücel D., Memiş A. (2010). The effects of N-acetylcysteine on testicular damage in experimental testicular ischemia/reperfusion injury. Pediatr. Surg. Int..

[143] Inceu A.I., Neag M.A., Craciun A.E., Buzoianu A.D. (2023). Gut molecules in cardiometabolic diseases: The mechanisms behind the story. Int. J. Mol. Sci..

[144] Crisóstomo L., Alves M.G., Gorga A., Sousa M., Riera M.F., Galardo M.N., Meroni S.B., Oliveira P.F. (2018). Molecular mechanisms and signaling pathways involved in the nutritional support of spermatogenesis by Sertoli cells. Methods Mol. Biol..

[145] Abd El-Fattah E.E., Saber S., Youssef M.E., Eissa H., El-Ahwany E., Amin N.A., Alqarni M., Batiha G.E., Obaidullah A.J., Kaddah M.M.Y. (2022). Akt-AMPKα-mTOR-dependent HIF-1α activation is a new therapeutic target for cancer treatment: A novel approach to repositioning the antidiabetic drug sitagliptin for the management of hepatocellular carcinoma. Front. Pharmacol..

